# The Genetic Code and RNA-Amino Acid Affinities

**DOI:** 10.3390/life7020013

**Published:** 2017-03-23

**Authors:** Michael Yarus

**Affiliations:** Department of Molecular, Cellular and Developmental Biology, University of Colorado, Boulder, CO 80309-0347, USA; yarus@stripe.colorado.edu; Tel.: +1-303-817-6018; Fax: +1-303-492-7744

**Keywords:** binding, triplet, codon, anticodon, DRT

## Abstract

A significant part of the genetic code likely originated via a chemical interaction, which should be experimentally verifiable. One possible verification relates bound amino acids (or perhaps their activated congeners) and ribonucleotide sequences within cognate RNA binding sites. To introduce this interaction, I first summarize how amino acids function as targets for RNA binding. Then the experimental method for selecting relevant RNA binding sites is characterized. The selection method’s characteristics are related to the investigation of the RNA binding site model treated at the outset. Finally, real binding sites from selection and also from extant natural RNAs (for example, the *Sulfobacillus* guanidinium riboswitch) are connected to the genetic code, and by extension, to the evolutionary progression that produced the code. During this process, peptides may have been produced directly on an instructive amino acid binding RNA (a DRT; Direct RNA Template). Combination of observed stereochemical selectivity with adaptation and co-evolutionary refinement is logically required, and also potentially sufficient, to create the striking order conserved throughout the present coding table.

## 1. Introduction

### 1.1. The Argument 

In all likelihood, construction of the genetic code required specific interactions between amino acids and RNAs, acting alone, before peptides could be encoded. Close study of this molecular interaction, therefore, is one of the most promising routes we possess to the origin of the code and translation itself. Here we test for unexpectedly frequent cognate coding triplets within, taking an essential role in, a specific set of RNA-amino acid binding sites.

### 1.2. Amino Acids as RNA Ligands

**Figure 1:**
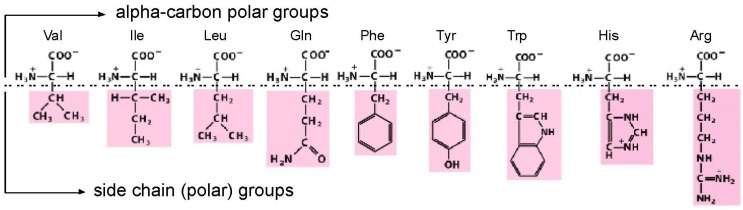
Amino acids studied by selection. The drawing divides the amino acids into two sites of possible interaction, divided by a horizontal dashed line. Firstly, α-carbon groups, which can make favorable polar interactions with RNA in every case. Then side chains, which can make stabilizing RNA contacts when their varied polar character allows. Val = valine; Ile = isoleucine; Leu = leucine; Gln = glutamine; Phe = phenylalanine; Tyr = tyrosine; Trp = tryptophan; His = histidine; Arg = arginine. His imidazole is drawn protonated, because the major His site [3] and the His-Phe peptide site as well [4] prefer protonated His imidazole.

Amino acids, though they are much smaller (MW ≈ 110) than nucleotides (MW ≈ 340), present two faces for interaction by nucleotides in RNA. As judged from crystal structures of riboswitches [1], RNA sites necessarily allow conserved, highly polar α-carbon groups (like carboxyl and amino) to be fixed in space by a convergence of highly directional polar interactions, such as hydrogen bonds [2]. With such a fixed, common foundation, an RNA binding site can also make bonds to a side chain group (Figure 1). This double-ended “polar profile” [1], of course, only applies to amino acids with two polar centers to offer. Further, even with possible bi-directional interactions in hand, other constraints (such as the selection for small site size) will favor interactions with one locus or the other.

For the purposes of biological structure and coding, we will be interested only in sites that include interactions with a side chain. Thus, relevant RNAs will bind both α-carbon/side chain or side chain only. Such sites are amino acid specific, and thus allow encoding of the amino acid. These distinctions are crucial to the function of RNA binding sites, because double-ended sites yield greater energies of interaction (∆G < 0) than single ended sites. For similar reasons, double-ended sites have greater stereoselectivities because they localize the sidechain (which transits the carbon tetrahedron when an l-amino acid becomes a d-amino acid). 

We can make these descriptions quantitative [1]. Based on 337 independently-derived binding sites for nine of the standard protein amino acids, potentially single-ended RNA sites have K_D_ from 10^−2^ to 10^−3^ M/∆G_bind_ −2.8 to −4 kcal/mol. The more intimately engaged amino acids, presenting two sites of interaction, have K_D_ from 10^−4^ to 10^−6^ M/∆G_bind_ from −5.5 to −8 kcal/mol at 25 °C. The stronger affinities are clearly consistent with several intermolecular bonds, to two sites.

Switching to stereoselectivity, apparently single-ended sites range from 1-fold (no distinction) to ≈30-fold (0–2 kcal/mol), and 10- to several thousand-fold (1–5 kcal/mol) in double-ended amino acid binding sites.

### 1.3. A Substantially Single-Ended Example Site, Isoleucine (Ile)

Notably, a less polar side chain does not rule out all amino acid selectivity. Hydrophobic sidechains like Val (valine) and Ile (isoleucine) (Figure 1) are of interest because they are observed in spark tube experiments [5], and are therefore thought of as primitive [6]. Despite these distinctions, they do not offer polar sidechain interactions. Nevertheless, an RNA site selected for l-Val [7] prefers it by 1.6 kcal/mol to l-α-amino-butyrate (one methylene group smaller). A site selected for l-Ile [8] prefers it by 0.82 kcal/mol to l-valine (one methylene group smaller). These findings raised the possibility of specific RNA bonds to aliphatic sidechains. However, these specificities are now instead believed to result from use of the size of the sidechain [1] as an essential site structural element, because further decreases in side chain size after removal of the first methylene have little effect.

### 1.4. A Frequently Double-Ended Example Site, Arginine (Arg) 

The arginine (Arg) side chain features a terminal guanidinium ion. The ion is planar, aromatic, positively charged, and offers a pattern of hydrogen bonding that matches the edge of nucleobases extremely well. This makes Arg sites very frequent in RNA; for example, such sites are smaller than sites for other amino acids [9]. In tallies of the content of RNA-protein interfaces, arginine provides the most numerous contacts [10]. This significance extends to regulatory interactions, where Arg contacts with RNA mediate regulatory modulation, for example, in the TAR peptide of HIV [11,12]. Further, Arg is unique in having unusual general interactions with folding RNAs, where it uniquely destabilizes tertiary folding, both slowing formation and speeding the breaking of a tertiary RNA contact [13]. For parallel reasons, it is no surprise that the first-detected specific amino acid binding site on RNA was for Arg [14]; the amino acid competes with G nucleotides for interaction with the splicing cosubstrate site on *Tetrahymena* self-splicing rRNA [15].

The ability of specific RNA folds to bind one or both amino acid domains will be a crucial point of discussion below.

## 2. The General Study of Amino Acid-RNA Binding by RNA

In order to generalize about amino acid-ribonucleotide interfaces, it is productive to study a number of them, involving bound amino acids of different types (Figure 1). The selection and cloning of RNAs [16,17,18] specifically eluted by cognate free amino acids [19] from carboxyl-immobilized amino acid columns provided this opportunity. Immediately above, some properties of binding sites obtained in this way have been listed. Below, I characterize the way the selection method produces its results to provide context for interpretation of now-numerous, newly-selected binding site sequences.

### 2.1. The Affinity Method 

Amino acids are immobilized at concentrations of several mM to tens of mM, usually by coupling their carboxyls to make amides, using amines linked to a chromatographic support via a neutral connecting arm. Large populations of randomized RNA sequences will contain some active amino acid sites that bind to such an immobilized amino acid. These bound RNAs can be eluted, after washing away unbound molecules, with solutions of amino acid. In effect, a small minority of RNA amino acid sites declare themselves by first becoming immobile on the fixed amino acids of the column, then being mobilized by the minor solution change produced by addition of a low concentration of, say, dissolved mM l-histidine (His) in column buffer. Because initial randomized sequences are usually flanked by constant sequences complementary to primers, RNAs that bind pure free l-amino acid, d-amino acid, or derivatives can be saved as DNAs, which are amplified, then transcribed from a promoter in a constant region to later use them.

Such affinity chromatographic procedures purify l-Ile-binding RNAs by ≈100-fold when first applied [20], typically declining to 1-fold (no purification) after five or six chromatography-amplification cycles, at which time ≈20% of transcripts are eluted by isoleucine.

### 2.2. Simple, Abundant Sites

It is vital to appreciate the target(s) detected by affinity selection. Experiments on the origin of the code do not seek sites with optimized performance, but instead, the simplest sites. That is, shorter RNAs that may exhibit less impressive affinities and selectivity [21]. This is because a primitive environment is likely to be restrictive to RNA synthesis and survival. Accordingly, the molecules most easily accessed, least sensitive to physical or chemical attack, seem the appropriate targets. 

That is—it certainly is possible to do selections that optimize a function. Selecting RNAs that slowly release a ligand selects most stable binding, for example, by l-Arg [22]. Alternatively, if a selection allows RNAs to compete for reaction at a limited number of sites, selection of the fastest reacting can be the result [23,24]. However, in the absence of such functional pressures, the most numerous RNAs, or most probable, or the simplest, are the ones readily isolated.

The latter case describes affinity selection. A 1 ml affinity column containing 1 mM ligand has 6 × 10^17^ potential RNA binding sites. Roughly 10^15^ total RNAs are added to initiate a selection, and a small fraction of these fold to produce amino acid binding sites. If 10^−10^ of random sequences have active sites [25], 10^5^ molecules of RNAs assort themselves among 6 × 10^17^ loci. Competition is vanishingly rare, even after selection has greatly increased the active RNA fraction.

Using equations for affinity chromatography at equilibrium [26], it can be shown [19] that a ‘typical’ column affinity selection recovers RNAs with K_D_ ≤ approximately half the eluant concentration; K_D_ ≤ 2.5 mM for free ligand when RNA is eluted with 5 mM ligand. This ability to examine simple RNAs with affinities into the mM range is another of the qualities that specifically suit affinity chromatography to coding studies.

#### 2.2.1. Number of Essential Nucleotides 

Usually, one can define nucleotides essential to RNA site functions using straightforward biochemical criteria. Such nucleotides are conserved in independent isolates; protected or sensitized to chemical probes by interaction with specific RNA ligands; or alter RNA activities if they are previously altered chemically or by mutation (e.g., [27]). The biochemically defined active site is the sum of such functional nucleotides, the number of “Implicated Site Nucleotides” (ISN). Implicated Site Nucleotides differ from the constellation of atoms also called nucleotides by a structural biologist, and sometimes the distinction is essential.

Though usually obvious, site nucleotides can occasionally be elusive. In the simplest l-tryptophan (Trp) site [28], a G flanking the amino acid binding loop is absolutely required for function, but so variable in position and in surrounding structure that it was not evidently conserved, and so was not initially detected [29]. Nevertheless, such cryptic requirements still affect the frequency of Trp-binding activity. For purposes of thought, the simplest l-His RNA site contained a mean of 20.1 ISN [4], the sufficient l-Trp site about 18 ISN [29], and the simplest l-phenylalanine (Phe) site 17.5 ISN [4].

In 1 A_260_ of the above partially randomized RNA (with flanking constant sequences), all contiguous 24-mer sequences will likely be present [19]. Shorter chains of essential nucleotides will be multiply present, and are more likely to be recovered. Thus, a simple summary is: the shortest contiguous sequences, usually having ≤24 essential nucleotides, should be the most likely to be isolated. These 24 “essential nucleotides” are defined by statistics. If only purines occur at a given position, this is twice as likely to occur as one specific nucleotide. In this case, selection can isolate twice as many such “essential nucleotides”. 

To put these ideas in another useful way, increasing the scale of an experiment by using 10-fold more RNA usually provides access to 1.66 additional essential nucleotides [19]. Thus, there are two kinds of selection experiments. One can do large experiments to seek large active motifs, but this usually implies looking among sparsely sampled molecules, because not all sequences of long lengths are present. Alternatively, one can look for smaller motifs, using RNA populations that contain many copies of them. Such an experiment tests every possible sequence of shorter length for the selected activity, which is often desirable. Increasing the amount of RNA moves the size boundary between these two experimental goals, 1.66 nucleotides for every 10-fold in RNA. This quantitative argument therefore also bears on the scope of small experiments. Because, typically, essential nucleotides ≤ ISN, selection experiments of practical laboratory size, even small ones, easily recover RNAs with enough ISN to fold functional amino acid binding sites.

#### 2.2.2. Modularity 

However, real RNA active sites are not usually made of the contiguous essential nucleotides discussed above. An active internal loop, for example, may be composed of two active ‘single-stranded’ loop modules which combine to yield an active two-sided loop surrounded by helices—with little regard to the initial spacing between the conserved loop modules. This is very important to real tertiary structures because the more modules, and the more even their sizes, the more ways there are to place them—thus the more frequently they occur within a randomized sequence [30]. Therefore, being composed of many pieces, in the best case pieces of similar size, can also determine whether an RNA structure can be isolated. Selections tend to isolate the most modular structures, as well as the ones containing the fewest essential nucleotides [31].

The reasoning that makes modules helpful also suggests that space is similarly good. Longer RNAs for selection should have more ways of, and be more capable of, manifesting a structure. To an extent, this is true experimentally; up to ca. 60 randomized nucleotides, the Ile RNA binding site becomes more frequent [20]. However, then in violating theory, it is less frequent in longer molecules. Perhaps long RNAs go to Uhlenbeck’s alternative conformer hell [32].

#### 2.2.3. Partially Conserved Nucleotides 

Even nucleotides not usually defined as conserved must be recruited to form an active site, like those that form variable paired regions around a more conserved internal loop. These requirements also reduce the frequency of sites, and can be subtle. 

For example, complementary primer sequences reduce the frequency of the prevalent Ile-binding site ca. 7.5-fold [20]. This effect can be traced to a displacement of site-bounding helices. The preexisting constant helical structure favors one permutation of the Ile site, because one bounding helix is easier to form from random sequences, thus also decreasing the accessible sequence space and total frequency of the Ile motif.

Adding site-defining stable helices to flank active Ile loop modules decreases active site occurrence by orders of magnitude [33]. The result is that about 4.1 × 10^9^ 100-mers or 0.2 nanograms or 7 femtomol of RNA chains must be searched to find the folded Ile-binding RNA. Judging from folding calculations, inhibitory folding effects appear to be a much smaller impediment than effects of the rarity of these bounding helical structures themselves. Nevertheless, these populations are orders smaller than the usual laboratory selection experiment. They therefore suggest that an RNA world with amino acid binding RNAs is more accessible than intuition at first suggests. 

#### 2.2.4. Constant Promoter/Primers 

The above Ile effect introduces the effects of flanking sequences, which can become directly or indirectly involved in the active sites. Such direct effects of constant sequences are easily found. Flanking sequences can be incorporated as ISN, thereby changing the most likely site. The incorporation of an AAA run from constant sequences completely changed the outcome of a selection for Ile-binding sites [34], reducing the most frequent motif in any other selection to a minority. Two-nucleotide constant tag sequences introduced for another reason led to isolation of a previously unseen motif for d-His binding [35]. When the unique tags were not supplied, the novel site did not appear at all in later selections.

As might be expected, the effects of constant sequences fade as the random region is lengthened, and the selected site (for Ile; [20]), on average, moves away from constant influence. However, the goal of coding experiments is to persuasively eliminate outside effects on the selection. This kind of spurious effect can be eliminated by re-isolation of the same site in the context of different constant sequences. For example, this has been done for the simplest Ile, His, and Trp-binding RNA motifs. A more specific strategy, for coding studies, is to bar an amino acid’s codons and anticodons from fixed sequences (and thus bar them from inducing complements in selected sequences), as was done for l-His [3,35]. 

Thus, a selection experiment also selects the constant sequences in the RNA transcript. Usually, this is of no concern. However, in a rare case the end(s) of the RNA are crucial to activity, and the RNA selected can change dramatically when a bounding sequence is changed or eliminated [36].

### 2.3. Sequentially Squeezed Selection 

As this discussion shows, many factors alter the occurrence of a selected RNA sequence. To simplify selection outcomes, and make them more easily interpretable, amino acid binding selections have been conducted in random regions of decreasing size. For example, l-Ile binding was sought within 26, 22, and 16 contiguous randomized nucleotides [25]. This size range is narrow enough to avoid size selection based on slower replication of longer molecules [37]. Moreover, the experimental design accentuates two well-known benefits. First, short RNA populations contain sequences, like the Ile binding site, at frequencies close to calculated from probability, whereas long RNAs are deficient [20]. Second, as pointed out above, short sequences can be fully represented in initial selection populations, so that RNAs derived are plausibly the only functional ones existing at that size.

As we hoped, one Ile site sequence was prominent at larger lengths, the majority sequence with selected activity at a shorter length, and then disappeared, leaving no *bona fide* l-Ile-binding RNAs at the shortest length. Thus, there is a predominant active structure, which persists as space for it is shortened. Squeezing appears to establish a reliable limit—when selection requires more nucleotides than randomized tracts provide, no shorter site is selected. 

Related experiments apparently yield the simplest amino acid site for l-Ile [20,25], l-His [35], l-Trp [28,29], and l-Arg [9]. This is not trivial in any case, but arginine is especially interesting. l-Arg-RNA interactions are unusually strong and versatile (see above). Thus, numerous l-Arg sites had been isolated. However, despite repeated selection, no l-Arg binding site had been observed more than once. Nevertheless, under sequentially squeezed selection, a simplest l-Arg site emerged. Note particularly that the shortest, simplest site in these experiments is required to be sidechain-specific (otherwise an amino acid cannot be meaningfully encoded). Thus, a squeezed specific selection probably focuses the site profile toward sidechain features.

Moreover, study of two activities side-by-side allows investigation of which is the simpler RNA function (takes place in the smaller site). Simultaneous mixed squeezed selection of affinity for d-His and l-His attached to a non-chiral glass support suggests that d-ribose RNA has an intrinsic chiral preference. It folds the simplest site for l-His using about one less essential nucleotide than required for the simplest d-His site [35]. The simplest l-His site was the same one [3] previously isolated for l-His alone using a different column matrix, different fixed sequences and solution conditions, strengthening the argument for selection of simple sites. This same chiral l-His RNA site has been taken through the looking glass, by synthesizing a Spiegelmer containing l-ribose rather than d-ribose. Ruta, et al [38] confirm that an enantiomeric switch in ribose also switches the RNA binding site to favor d-His.

### 2.4. Reproducible Selections 

I emphasize a general conclusion about amino acid affinity selections. Within appropriate limits, for example, attributable to the need for fixed flanking sequences that do not intrude, selections have a predictable outcome. There are, reproducibly, simplest sites. The simplest l-Ile site has been independently isolated 267 times [20]. Even for a versatile amino acid like l-Arg, which binds quite variable, small ribonucleotide sequences—nonetheless a properly constrained search repeatedly finds particular simple, recurring binding sites [9]. By extension, given predictable selection, evolution at the amino acid-RNA level of complexity can be productively interrogated by experiments, and reliable relations between amino acids and RNA sequences can be derived. 

## 3. Amino Acid Binding Sites and Coding Triplets 

**Figure 2:**
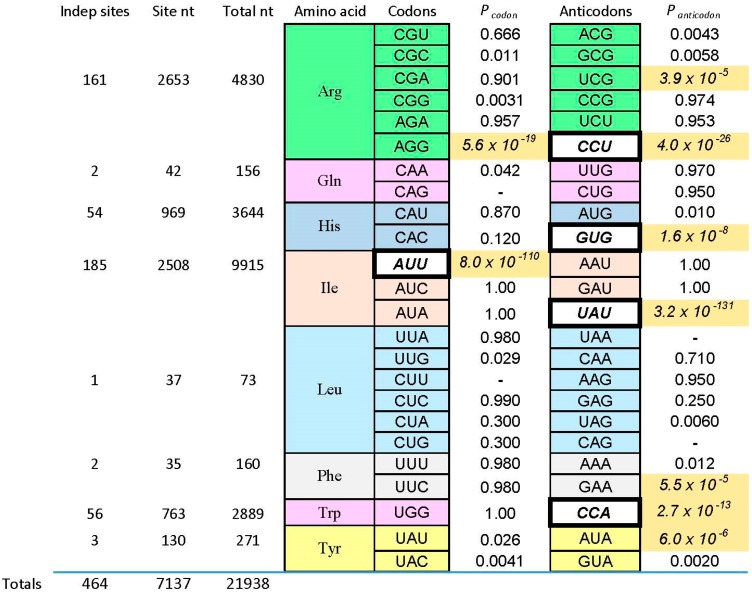
Probability of uniform distribution of codon and anticodon triplets. Here the Implicated Site Nucleotides (ISN) are compared with other initially randomized positions in individual selected amino acid binding RNAs. Fixed sequences, of course, are not considered. Under the null hypothesis that cognate triplets (listed in columns) are equally frequent inside and outside the ISN, the probabilities of equal triplet distributions for eight kinds of amino acid sites are tabulated. Probabilities come from a two-tailed G test with Williams correction [39]. Probability boxes containing dashes are triplets that did not exist in the experimental sample. Among probabilities, shaded boxes with italicized numbers are significant. To evaluate significance in a conservative way, I compute P_sig_ = 1 − (1 − P_err_)^1/n^ where P_sig_ is the maximum acceptable probability and P_err_ is the target error for each of the n trials in the Figure. To limit the probability of error to P_err_ = 0.01 in 44 individual trials, the maximum probability regarded as significant is P_sig_ = 2.3 × 10^−4^. Among triplets, italic triplets on white backgrounds are those concentrated by sequential squeezed selections for the cognate amino acid. Binding and sequence data can be found in: Ile [8,20,25], leucine (Leu) (I. Majerfeld, M. Illangasekare, M. Yarus, unpublished; see [1], Gln (C. Scerch and G. Tocchini-Valentini, pers. comm; see [1]), Phe [40], Tyr [41], Trp [28,29], His [3,35], Arg [9,22,26,42].

We now consider one of those “reliable relations” in selected RNA-amino acid binding sites. What follows (and what came before) is based on data for eight amino acids of varied chemical classification (Figure 1): charged polar (Arg^+^, His^+^), uncharged polar (Tyr, Gln), aliphatic hydrophobes (Ile, Leu) and aromatics (Phe, Trp). The survey is partial, but quite broad (Figure 2). There are 464 independently derived sites in the characterized populations, Implicated Site Nucleotides number 7137, and total nucleotides, inside and outside amino acid sites, are 21,938. Tested amino acids emerged from the evolution of the code with six, three, two, and one triplet(s). Site sequences have been examined for 44 coding triplets altogether, 22 cognate codons and 22 cognate anticodons. The results surveyed are those referenced earlier [1], updated for the sequentially squeezed selection for l-Arg [9].

*P_codon_* and *P_anticodon_* are probabilities that the associated coding triplets are equally frequent outside each site and inside (within the ISN of) each site. That is, Figure 2 tabulates the probability that frequencies outside and inside are equal, by the G test—related to Chi-squared, but more versatile [39]. Equality is not the rule, as shown by probabilities with triple-digit negative exponents observed in Figure 2. Instead, seven cognate anticodons and two codons are very significantly elevated (marked by shaded backgrounds for probabilities) in the ISN that are most closely connected to a bound amino acid. The control is initially randomized nucleotides also in the selected RNAs, also selected using the same procedures, but outside the ISN of the active binding site. Further, coding triplets in boldly outlined white boxes in Figure 2 are the one codon and four anticodons concentrated in sequentially squeezed selections for RNAs binding Trp [29], His [35], Ile [25], and Arg [9]. No amino acid site concentrates codons alone; real cases either present both codons and anticodons (Arg, Ile) or anticodons alone (His, Phe, Trp, Tyr). Notably, the positive results can be called sparse: only two of 12 Arg triplets are significantly implicated by selected sites, or two of six triplets for Ile. Other cases have found only one triplet concentrated in RNA binding sites. Sparseness is a crucial finding, whose implications reappear below.

**Figure 3:**
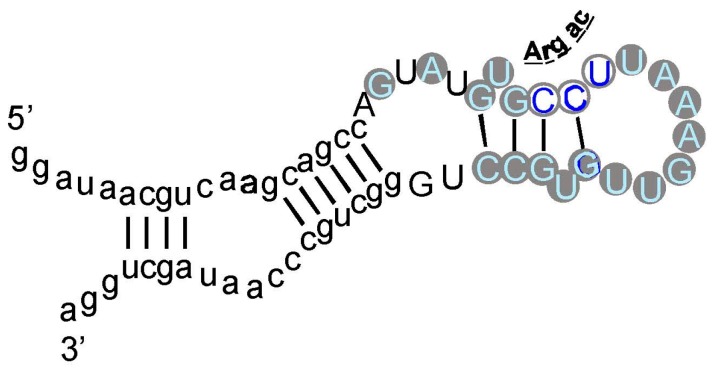
An example: the most prevalent Arg-binding RNA. Arg-606 [9], derived from a 25-nucleotide randomized region, is shown. Lower-case letters are fixed sequences, capital letters represent originally randomized nucleotide positions. The nucleotide sequence is threaded through the probable secondary structure for all related isolates, deduced by BayesFold [43]. Gray circles mark Implicated Site Nucleotides, and the three open gray circles are a very highly conserved arginine anticodon (cognate to codon AGG). Arg-606 had K_D_ = 0.5 mM, and D/L ≈ 35, consistent with the idea that the smallest sidechain-specific sites are predominantly single-ended. Comparable simplest His, Ile, and Trp sites from separate sequentially squeezed selections have been reviewed [27].

As an example, an arginine site is shown in Figure 3, where one of the most prevalent l-Arg binding motifs is drawn. Gray circles mark Implicated Site Nucleotides. RNAs closely related to this one, which bind l-Arg near the junction of a short helix and a highly-conserved 8-membered hairpin loop (Figure 3), comprised 62% of all isolated RNAs. Related small sites conserve the l-Arg anticodon marked at the entry to the hairpin loop (Figure 3) in 94% of all sequences. These motifs are well-represented even when given only 17 initially randomized nucleotides to fold. 

## 4. Tiny Probabilities 

Below, I argue that minute probabilities in Figure 2 are reliable guides—cognate coding triplets are improbably elevated within RNA binding sites. These particular minute magnitudes are produced by the experimental context. Sequentially squeezed selections generate many new, independently derived binding sites. If a conserved cognate triplet appears in the simplest site, more sites with this non-random outcome force the probability of an unbiased distribution progressively down. This is evident in Figure 2, where the tiniest *P_codon_* and *P_anticodon_* are in white boxes associated with squeezed selections. However, that being said, what of it? This behavior characterizes any true hypothesis. The more experimental evidence, the less probable that we will contradict a true finding. Moreover, Figure 2 contains cases like Phe and Tyr, where characterization of a few motifs from a normal selection turn up an improbably concentrated cognate triplet. This was true for sequentially squeezed selections also, before they were squeezed. Therefore, association of cognate triplets with RNA binding sites does not depend on a special experiment—it was evident, in all cases, among initial examples isolated.

### 4.1. Observed Triplet Concentration Is Not Attributable to the Statistical Test 

The test used in Figure 2 (G test for goodness of fit with the Williams correction [39]) is related to one universally used to test ratios in genetic crosses, and is therefore employed widely in Biology. However, no test, nor any assumption whatever about the natural distribution of triplets within RNAs is needed to reach the conclusion that the null hypothesis (triplets equivalent everywhere) is very improbable. For l-Arg [9], nucleotide sequences of isolated RNAs were randomized 10^6^ times, and the resulting “binding sites” at previous positions were retested. The concentration of the Arg CCU anticodon in real binding sites, for example (Figure 2 and Figure 3), was not observed in a million such tries.

### 4.2. Triplet Concentrations Have the Logic of Real Coding: Reversed Triplets 

5′ to 3′ reversed codons (e.g., UUC Phe > CUU) and anticodons have the same compositions and the same predicted random frequencies as true triplets. Such reversals would be concentrated in binding sites by any accidental process. Moreover, if binding sites (or nonbinding sites) express an underlying preference for certain nucleotides or triplet compositions, reversed triplets would succumb. Thus, it is striking that, tested for multiple RNAs binding each of six amino acids, multiple observed excesses of cognate triplets of both kinds vanish when tested triplets are reversed [44]. Because binding sites contain several triplets (compare Figure 3), one might argue that at a significant frequency, cognate triplets will recur by chance. Evidently, this is rare, since reversed codons and anticodons do not observably do so, given 42 triplets evaluated in 22 site sequences of six specificities.

### 4.3. Triplet Concentrations Have the Logic of Real Coding: Variation of the Code 

Fifty million randomized codes have also been tested for triplet localization in experimental binding sites [44]. Notably, 10^6^ new codes were derived in five ways: with codons placed randomly in the Coding Table, amino acids assigned randomly among real coding blocks, amino acid identities assorted to blocks of the same size, randomization of triplet position 1 and 2, and reassignment of initial codon doublets. In short, 99.2% to 99.5% of these randomized codes give less association with observed binding sites than the real code, and those that do yield association tend to be those retaining fragments of initial code structure. There are important positive and negative implications. Positively, triplet excesses in experimental RNA binding sites are strongly associated with assignments made during evolution of the bona fide coding table. Negatively, these data are further strong evidence against accidental links between triplets and cognate amino acids as a result of these procedures (Figure 2). 

### 4.4. Relation to Natural Cases 

Remarkably, pooled experimental results in Figure 2 overlap evidence from natural RNA sequences. The *Tetrahymena* self-splicing group I intron binds arginine [14], and guanidinium ion as an analogue of the Arg side chain, using the G of a conserved Arg codon AGA/CGA/AGG [45]. Arg guanidinium (terminus of the Arg side chain in Figure 1) emulates the base-pairing of G, so it can bind at the same site [15,46]. Because this is within the active site for the co-splicing substrate, a guanosine nucleotide, Arg and guanidinium inhibit splicing [14]. This behavior overlaps the concentration of AGG triplets within newly selected RNA structures that bind Arg (Figure 2). Thus, the initial evidence which initiated studies of amino acid-RNA binding is echoed in present selection results.

An even more surprising case appears in riboswitches regulated by guanidinium ion, in bacteria that need to control its modification and export [47]. Riboswitches regulate linked messages by changing structure on binding metabolites. There are, for example, RNA riboswitch domains that bind Gly [48], Lys [49], and Gln [50]. Such RNAs usually have complex structures and functions, and so are not plausibly related to selected simplest amino acid sites. 

**Figure 4:**
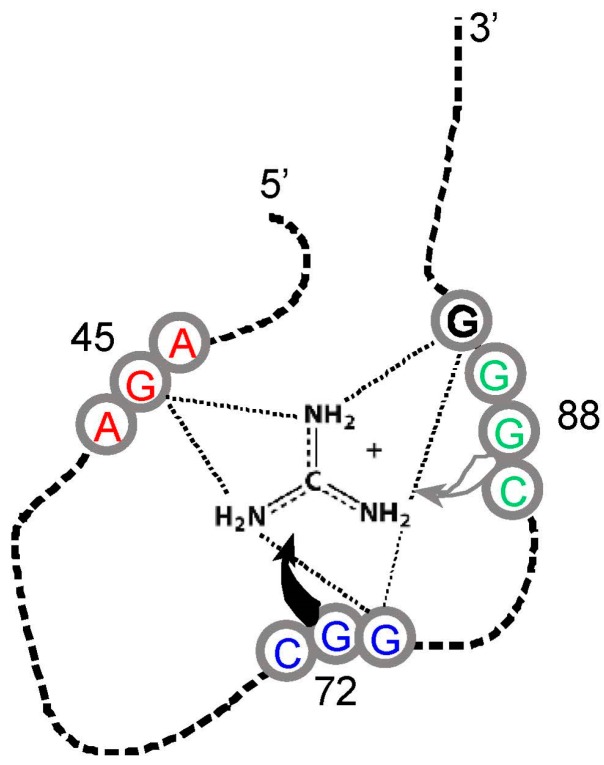
Schematic structure of a *Sulfobacillus* guanidinium riboswitch. Gray numbered circles are nucleotides of the crystallographic structure for guanidinium ion bound to the sensor of the guanidinium-I riboswitch. Dotted lines are hydrogen bonds; the gray and black curved arrows indicate that G72 covers the top, and G88 forms the bottom of the guanidinium binding site, respectively. Arg triplet nucleotides are colored; the ones centered at G45 and G72 are almost completely conserved; at G88, ≈75% conserved. G90 (black) is a non-triplet site nucleotide. Drawn from [51] and Protein Data Base (PDB) structure 5T83.

However, guanidinium ion may be an exception. This small-molecule analogue of the Arg side chain terminus (Figure 1) is bound within the conjunction of three conserved Arg codons, AGA/CGG/CGG (Figure 4). Nucleotides of the three Arg codons are not only in close contact with the ligand, but completely fill the space around guanidinium and engage all of the polar groups of the ion [51]. Using an adjacent G surface, this three-Arg-triplet site also includes close contact with the top and bottom of the Arg side chain analog. The *Tetrahymena* site binds Arg [14], though for lack of space, the *Sulfobacillus* site does not admit the complete amino acid [47]. However, both natural examples display extreme concentration on the distal amino acid side chain of Arg, accompanied by cognate coding triplets. Thus, sites in *Tetrahymena* rRNA and *Sulfobacillus* riboswitch aptamers suggest that for Arg, the chemical connection between arginine/guanidinium affinity and coding triplets has found biological uses which persist into modern organisms. Such contemporary interactions may be much more frequent—anticodons in rRNA appear appreciably concentrated close to cognate amino acid sidechains in four crystallographically defined ribosomes [52].

It is unexpected that an amino acid affinity purification isolates RNA sequences that repeatedly show a specific formal relation to the genetic code. Moreover, similar interactions for Arg appear in natural RNAs. These data (Figure 2 and Figure 3) are particularly interesting because squeezed sites, and natural RNAs that emulate squeezed sites by concentrating on the terminus of a side chain, also elevate the probability of essential coding triplets. In light of these repeated findings, there does not seem to be a plausible alternative to the conclusion that RNA binding sites recapitulate an essential event during the evolution of the amino acid code—but what event? Association of triplets and cognate sites is itself objectively demonstrable. However, to reason about the foundational events of the genetic code, a bit of speculation is required.

## 5. Direct RNA Templates (DRT) 

The apparent simplest way to use these findings in primordial translation uses an RNA template that directly binds (activated) amino acids side by side, so they subsequently react to form ordered, encoded peptides. This emulates the mechanism of the ribosomal peptidyl transferase itself—it accelerates its reaction principally by apposing reactants [53]. Cognate RNA triplets within amino acid binding sites subsequently evolve to act as anticodons in tRNAs and codons in mRNAs [1]. In fact, the potential co-occurrence of amino acid specificity, anticodons, and codons together in one RNA binding site is an intrinsically striking property. RNAs studded with multiple aminoacyl-RNA synthesis centers at a potential mean spacing of only a few nucleotides are also well known [54], and similar aminoacyl transfer centers can be supplied with activated amino acids by a ribozyme [55,56]. These data together make possible RNA encoded peptide synthesis resident in one small RNA complex. The advantages of DRT simplicity have been argued before [1], though there are other possibilities [57]. 

**Figure 5:**
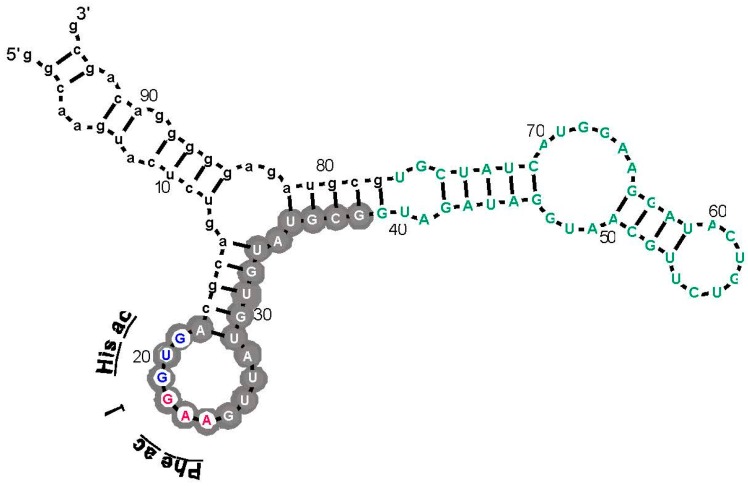
RNA 8, which has affinity for His and Phe in His-Phe. Lower case letters are fixed sequences, capital letters are initially randomized positions [4]. The RNA is threaded through the most probable secondary structure computed for its independent isolates by BayesFold [43]. Gray circles mark Implicated Site Nucleotides; those with white centers are potential coding triplets labeled “Phe ac” (ac = anticodon) and “His ac”. Green nucleotides are non-site, but initially randomized nucleotides.

Because we were interested in the molecular constraints on a DRT, we selected RNAs that bind [4] two amino acids in peptide linkage, NH_2_-His-Phe-COOH, retaining specificity for both side chains. His and Phe were used because their binding as free amino acids was already understood (see references, Figure 2). This experiment required counterselection against affinity for His and Phe individually, because singly-directed sites require fewer nucleotides. Thus, affinity for a single side chain (usually protonated His, Figure 1) is selected preferentially. When the census of ISN is taken on these sequenced and characterized His-Phe RNAs, His sites required 20.1 ISN, Phe 17.5 ISN, and His-Phe 24.4 ISN (averaging all RNAs in the two prevalent motifs for the latter). As an example, RNA 16 has K_D_ = 90 μM for His-Phe, 13 mM for l-His, and 100 mM for l-Phe. Thus, a peptide-binding RNA, even one that contacts both side chains, is not the sum of two amino acid affinities. Instead, the peptide site is only ≈35% larger than a site for one amino acid. Consistent with these counts, neither the previously known His site, nor the known Phe site, appear in these selected His-Phe RNAs. A new dual, smaller site is selected instead. An example of the most frequent His-Phe site is shown in Figure 5.

A ready rationale exists for smaller individual amino acid sites, still side chain specific. These can be extreme single-ended sites (see above), forced to be small because of the crowding of two sites produced by the short single covalent peptide bond between His and Phe. To be consistent, this kind of l-His site was not produced by sequential squeezed selection [35], so its structure must depend on the adjacent Phe residue or site. The existence of this kind of molecule supports the DRT, because it shows that RNA that binds DRT substrates (which are like free amino acids) can also bind the peptide product (His-Phe). Thus, for catalysis, only the binding of the transition state for peptide bond formation has not been shown, and this predicted activity can now be subjected to experimental search.

However, support for a DRT from this work has another, more surprising dimension. The sequence of the ISN for His-Phe RNA (Figure 5) contains adjacent His and Phe anticodons (white centers, Figure 5). Further, these are the same triplets over-represented in newly selected separate His and Phe binding sites (Figure 2). In this experiment, we do not have the statistical power (Figure 2) or structural resolution (Figure 4) of the general investigation of amino acid sites, whose interpretation presently relies on almost 100-fold more sites than for His-Phe peptide. Thus, caution is appropriate. Nevertheless, anticodon triplets (Figure 5) are noticeably conserved. There are seven independent parental molecules (12 isolates) of the His-Phe RNA shown. Three of seven have the Phe anticodon shown, two of those also have the adjacent His anticodon [4]. 

It would be unexpected to discover a new series of amino acid sites connected to the genetic code, in peptide binding sites. So, it is probably not a new set of sites, but simply a more radically squeezed structure. In other words, a partial site, not stable without the adjacent amino acid, but containing the same cognate anticodon as in the free amino acid site (Figure 2). This idea merits further investigation. Meanwhile, specific His-Phe peptide affinity, accompanied by individual sidechain contacts and cognate anticodons, are remarkably consistent with a primordial DRT. 

## 6. The Origin of the Genetic Code Is a Puzzle Whose Pieces Fit Together 

Two other major accounts of the code’s history, co-evolution [58], and adaptation [59], also have major roles to play. These roles are, in fact, now explicitly defined by data in Figure 2, in the following sense. 

Co-evolution is the idea that an early code ceded codons to later amino acids or acquired unused codons, as biochemical pathways extended the amino acid repertoire. This idea can be analyzed by comparing the coding table to biosynthetic pathways [60]. Adaptation theories propose that the code was created by optimization, most explicitly by reducing errors created by mistranslation [61]. Adaptation can be supported by showing resemblance between the genetic code’s order and an optimized arrangement on the basis of similar amino acid chemical properties [62].

Both co-evolution and adaptation require a pre-existing code. There must be coding to be extended as biosynthesis advances. There must be coding to be optimized by adaptation. Therefore, both hypotheses require something like the stereochemically-defined core suggested by RNA binding data (Figure 2). In one sense, this pre-existing stereochemical core is likely to be substantial. Six of eight arbitrarily characterized amino acids (Figure 4) concentrate their anticodons in the ISN of binding sites selected from random RNA sequences. Thus, excepting Gln and Leu, traces of a canonical core are observed for 75% of amino acids surveyed.

### 6.1. The Nature of the Stereochemical Basis 

However, 75% overstates the results in an important way. As pointed out above, coverage of the 48 possible triplets in binding sites is sparse. Arg is the high extreme: one of its six codons, and two of six anticodons are implicated by selection results (Figure 2 and Figure 3). If one adds the group I self-splicing RNA [45], the count rises to three Arg codons and two anticodons. Provisionally adding the guanidinium specific riboswitch site yields one new codon [51]. Thus, this extensive dataset yields associations with six of 12 possible Arg triplets. Moreover, in the more complete survey (Figure 2) of eight amino acids, 12 of 48 possible associations have been detected. As Arg surely illustrates, we can be surprised by new data. However, it is more plausible that exacting chemical requirements for participation in a specific RNA binding site’s tertiary structure can only be satisfied by a few cognate triplets, of all those available. The final result might be estimated close to the current average for eight amino acids, 25%, and less than the maximum 50% of triplets for Arg, the most RNA-accessible amino acid. That is: given present accounting (Figure 2, Figure 3, Figure 4 and Figure 5), the majority of triplets may have entered the code another way, rather than via RNA-amino acid specificity.

### 6.2. Co-Evolution Is Needed to Reach Barren Areas 

This reasoning implies a role for co-evolution and adaptation. How might one extend coding to triplets not touched by amino acid sites, like those for Gln (Figure 2)? A clear possibility is: one can co-evolve to adopt them. In fact, it has been suggested [63] that the existence of Glu-tRNA^Gln^, a modern metabolite and possible co-evolutionary intermediate in the incorporation of the Gln codons, is strong support for co-evolution to Gln coding. This Glu-tRNA^Gln^ argument also complements negative RNA binding evidence for Gln triplets from selection (Figure 2).

### 6.3. Adaptation Is Needed to Fill Boxes 

As for adaptation: how might one fill in the six kinds of partially occupied coding boxes sparsely created by RNA affinities (Figure 2)? The logic of RNA binding sites has no apparent reason to respect the neat groups of six or four or three or two triplets so characteristic of the code. However, this is easily rationalized as the result of a process which minimized the effect of translational ambiguity by evolving to use sets of related triplets. In fact, it can be shown that even levels of pre-existing stereochemical assignment we have found still allow resulting codes to be optimized [64]. There is no logical inconsistency in believing both stereochemistry and adaptation were influential in code history. 

## 7. Conclusions

We decisively confirm the hypothesis in this review’s first paragraph. The RNA-amino acid interface does contain the logic of (some of) the genetic code, relating triplets to amino acid side chains. Cognate triplets, though their functions may vary, are unexpectedly close to their amino acids. The conclusion is unequivocal—the probability that the contrary is true hovers in negative exponential triple digits (Figure 2, Figure 3, Figure 4 and Figure 5). These data together strongly confirm intuitions of Crick [65], Orgel [66], and Woese [62], who thought that such a connection would exist. 

It is presently less clear how to incorporate this finding into the code’s history, but early data on a Direct RNA Template are very positive (Figure 5). Among the most probable His-Phe RNAs are frequent molecules contacting both amino acid side chains, held at a spacing appropriate to peptide synthesis, and containing both cognate His and Phe anticodons.

Accordingly, events attending the birth of the genetic code are still remarkably evident in modern RNAs and amino acids. This implies that modern molecules are very similar to their ancestors. This is consistent with the tree of life on Earth [67], which shows that the code and translation are virtually universal, so their molecules trace back at least to the Last Common Ancestor [68]. In the experiments above, we show that these agents are older yet, likely surviving from the first encoded ancestral peptides. This is crucial data; modern biochemicals are tacitly assumed relevant in many studies of molecular evolution.

Finally, though study of the route to the full code is just beginning, several strong constraints have empirical support (Figure 2). Despite persuasive evidence for cognate triplets in RNA binding sites, neither the resulting stereochemistry, nor adaptation, nor co-evolution are plausibly sufficient to create the entire code, acting alone. Stereochemical affinities are uniquely capable of initiating coding, but extension of such initial assignments via co-evolution and adaptation are probably essential to complete the modern coding table.
